# Emotional intelligence and burnout in youth athletes: the mediating roles of sleep quality, fatigue, and delayed onset muscle soreness

**DOI:** 10.3389/fpsyg.2025.1719916

**Published:** 2025-12-03

**Authors:** Yingcheng Huang, Hongqian Liu, Guanhua Zhu

**Affiliations:** 1 Department of Sports Science, Guangzhou College of Technology and Business, Guangzhou, China; 2 Academy of Music & Guizhou Aerospace Vocational and Technical College, Guizhou University, Guizhou, China; 3 Olympic Institute for Advanced Study, Beijing Sport University, Beijing, China

**Keywords:** emotional intelligence, sleep quality, fatigue, delayed onset muscle soreness (DOMS), burnout, youth athletes, team sports, individual sports

## Abstract

Youth competitive sports expose athletes to substantial physical and psychological demands, yet the mechanisms through which physiological stress translates into psychological burnout remain insufficiently understood. Existing research has seldom examined emotional intelligence (EI) together with key recovery-related variables such as sleep quality, fatigue, and delayed onset muscle soreness (DOMS), nor has it clarified whether these relationships differ across sport types. Drawing on data from 450 collegiate athletes, this study investigated the combined and moderating effects of EI, sleep quality, fatigue, and DOMS on burnout, and compared these pathways between team and individual sports. Results showed that sleep quality served as a protective factor against burnout (*β* = −0.21, *p* < 0.001), whereas fatigue (*β* = 0.31, *p* < 0.001) and DOMS (*β* = 0.31, *p* < 0.001) were strong predictors of psychological exhaustion. EI consistently buffered the effects of these physiological stressors (*β* = −0.09 to −0.14, *p* < 0.01), indicating that athletes with higher EI preserve psychological resources more effectively under physical strain. Multi-group analysis revealed that fatigue and DOMS were more influential in team sports, while the protective role of EI was more pronounced in individual sports. These findings extend current psychophysiological models by demonstrating that burnout arises from both excessive physical load and insufficient psychological resources. The study concludes that recovery routines alone are unlikely to prevent burnout and highlights the need to integrate EI development with systematic load management and sport-specific support strategies to promote both performance sustainability and long-term mental well-being.

## 1 Introduction

**Figure 1:**
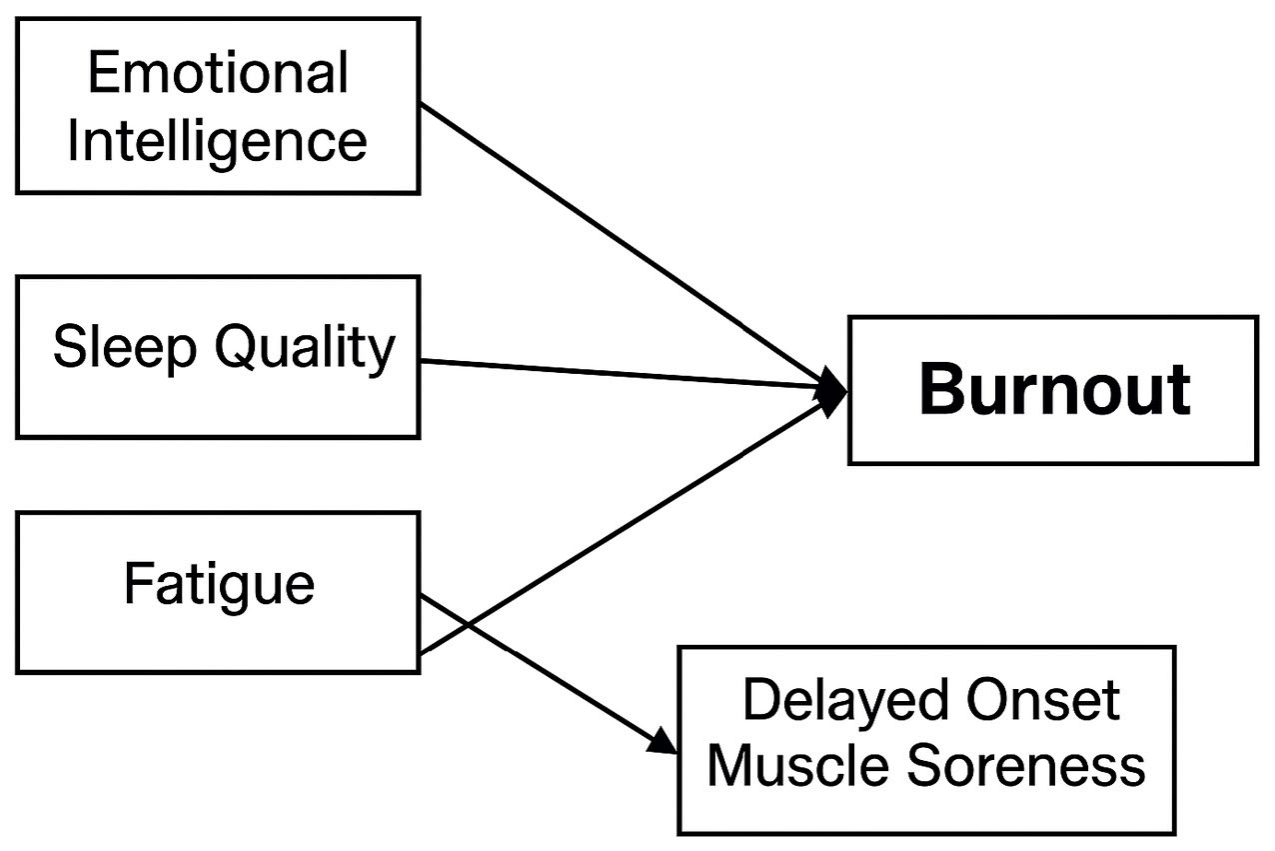
Psychological and physiological factors in adolescent athlete burnout.

Over the past two decades, youth competitive sport has increasingly been recognized as a complex developmental setting in which physiological demands and psychological pressures interact continuously. Cross-regional studies from Europe, North America, East Asia, and Oceania indicate that emotional intelligence, sleep quality, training-related fatigue, delayed onset muscle soreness, and athlete burnout are closely interconnected rather than isolated constructs. High-volume and high-intensity training often disrupt sleep rhythms, reduce deep sleep duration, and prolong sensations of fatigue and muscle soreness, thereby lowering recovery efficiency and increasing the likelihood of starting subsequent training sessions in a depleted state. Disturbed sleep and accumulated fatigue further weaken emotion regulation and attentional control, making young athletes more reactive to performance pressure, intra-team competition, selection mechanisms, and academic demands. These psychological disruptions reduce training engagement, undermine self-efficacy, and heighten the probability of developing burnout symptoms. Research also shows that emotional intelligence plays a pivotal role within this chain of processes. Athletes with higher emotional intelligence tend to recognize emotional and bodily signals more accurately, respond to stress and physical discomfort with more adaptive strategies, and maintain greater psychological stability under similar training loads. Those with lower emotional intelligence are more likely to interpret fatigue or tension as signs of failure or uncontrollable stress, amplifying negative emotion and slowing psychological recovery. Existing evidence suggests that changes in sleep, fatigue, and soreness can be viewed as a physiological stress pathway shaped by training load, while emotional intelligence influences how athletes perceive and regulate these stressors, thereby modifying the speed and magnitude with which physiological strain develops into burnout. Whether young athletes experience emotional exhaustion, reduced accomplishment, or sport devaluation depends on the long-term interaction between physiological load and psychological resources. Clarifying these relationships is essential for understanding the mechanisms underlying burnout and for designing effective training and recovery strategies. To synthesize these relationships, Figure 1 presents the conceptual structure linking physiological load, psychological resources, and burnout among adolescent athletes.

Although research examining the relationships among sleep, fatigue, and burnout in adolescent athletes has expanded considerably, the existing evidence still presents several notable limitations. Much of what is known comes from single-sport or narrowly defined samples—for example, Watson et al. (2022) focused on volleyball players, Dai et al. (2024) examined youth soccer athletes, and Chou et al. (2021) studied basketball players—leaving open the question of whether sleep patterns and fatigue responses generalize across different sport disciplines. Methodologically, current studies rely predominantly on cross-sectional designs (Gomes et al., 2017; Yan and Wang, 2025), making it difficult to trace how sleep, mental health, and burnout change over time; the few longitudinal studies that do exist (Grewal et al., 2024; Wingerson et al., 2021) are based on small samples, often composed of elite or adult athletes, which limits the ability to draw firm conclusions about developmental trajectories in youth populations. Theoretical work in this area is likewise fragmented. Existing studies typically examine discrete associations—such as the relationship between sleep and well-being (Watson and Brickson, 2018; Stavrou et al., 2021), the link between training load and fatigue (Aloulou et al., 2021; Tate et al., 2025a), or the developmental course of burnout (Saarinen et al., 2025)—yet they rarely consider the potential moderating role of emotional intelligence. As a psychological resource central to emotion regulation and stress management, emotional intelligence may attenuate the negative effects of poor sleep and accumulated fatigue on burnout, but this possibility has not been empirically tested. Research on recovery and intervention strategies is dispersed in a similar way. Prior efforts have explored acupuncture-based treatments (Luetmer et al., 2019), nutritional and recovery protocols (Mason et al., 2025), and temporary training adjustments during the COVID-19 pandemic (Wingerson et al., 2021), yet these studies tend to be short-term or tied to specific contexts, offering limited insight into broader mechanisms. Moreover, explanations of burnout continue to diverge along separate theoretical lines. Physiologically oriented studies emphasize the consequences of escalating training loads, showing that sleep disturbance, fatigue accumulation, and pain episodes undermine emotion regulation and cognitive control, thereby accelerating exhaustion (Stracciolini et al., 2019; Nakagi et al., 2024). Psychologically oriented studies highlight the role of emotional intelligence in emotional monitoring, stress appraisal, and social functioning, noting that athletes with higher emotional intelligence often employ more flexible and adaptive regulation strategies (Isoard-Gautheur et al., 2015; Markati et al., 2019). Yet physiological studies seldom incorporate psychological resources, while psychological studies rarely assess physiological indicators, resulting in two largely parallel streams that seldom converge. Attempts to integrate both domains have begun to appear, but their generalizability remains limited due to constraints related to sport type, cultural context, or sample composition. Taken together, the field still lacks a unified framework capable of explaining how physiological stress processes and psychological adaptation jointly contribute to burnout in adolescent athletes, and this fragmented body of evidence continues to restrict deeper theoretical development and limits efforts to build a comprehensive understanding of how burnout emerges.

These limitations highlight the need for a more comprehensive analytical approach that captures the combined influence of physiological stress and psychological adaptation in youth athletes. Existing studies seldom incorporate sleep quality, training fatigue, and delayed onset muscle soreness into a unified framework alongside emotional intelligence, and even fewer examine whether these relationships differ between team and individual sport contexts. Variations in training rhythms, social structures, and emotional demands across sport types may shape how physiological and psychological factors jointly contribute to burnout, yet these contextual distinctions have received limited empirical attention. To address these gaps, the present study develops an integrated framework that simultaneously considers emotional intelligence, sleep quality, fatigue, and muscle soreness, and tests whether the mechanisms linking these variables vary across team and individual sports. In doing so, the study advances current knowledge in three ways: it incorporates key psychological and physiological indicators into a unified analytical structure; it compares athletes from different sport types to clarify how contextual characteristics shape these interrelations; and it introduces a dual-dimensional perspective that offers new empirical and theoretical insight for improving training, recovery, and mental-health practices in youth sport settings.

Building on these gaps, the present study contributes to the literature in three primary ways:

*H1*. Poor sleep quality, greater training fatigue, and higher levels of muscle soreness are positively associated with burnout in adolescent athletes.

*H2*. Emotional intelligence is negatively associated with burnout and moderates the effects of sleep quality, training fatigue, and muscle soreness on burnout, such that these physiological stressors have weaker effects among athletes with higher emotional intelligence.

*H3*. The relationships among emotional intelligence, sleep quality, training fatigue, muscle soreness, and burnout differ between team-sport and individual-sport athletes.

## 2 Review

### 2.1 Current research on sleep and recovery in adolescent athletes

Sleep is a cornerstone of both physical and psychological recovery, exerting a profound influence on athletic performance and overall well-being in youth athletes. It has long been recognized as a central topic within sport psychology and sports medicine. According to Watson et al. (2022), the degree of sport specialization among adolescent female volleyball players was closely linked to sleep quality, which in turn shaped their overall life satisfaction and sense of well-being. Likewise, Stavrou et al. (2021) found that inadequate sleep markedly reduced alertness and perceptual functioning in both adolescent and adult athletes. Supporting these observations, Dai et al. (2024) demonstrated that insufficient sleep directly impaired match performance among youth soccer players in club environments. These findings align with a broader consensus that sleep is essential for both acute recovery and long-term athletic development, as emphasized in recent reviews on sleep and youth athletes (Coel et al., 2023; Pujalte and Benjamin, 2018). A systematic review by Nakagi et al. (2024) further highlighted that poor sleep quality significantly increases the risk of sport-related injuries. When considering group differences, Jaiswal and Shashikala (2020) reported that adolescent athletes generally demonstrated better sleep quality than their non-athlete peers, suggesting that consistent training may foster healthier patterns—although this benefit is not universal. Extending this line of evidence, Grewal et al. (2024), in a longitudinal study of professional cricket players, showed that sleep relates not only to physical recovery but also to emotional state and mental health. Seasonal variations have also been documented: Chou et al. (2021) noted that during competitive phases, elite basketball players experienced noticeable declines in sleep quality along with increased fatigue. Corroborating these seasonal patterns, Potter et al. (2020) and Sawczuk et al. (2021) found that youth athletes’ perceptions of sleep strongly predicted their general well-being and fatigue across training cycles. Mason et al. (2025) further pointed to the interconnectedness of sleep, recovery, and nutrition, observing that adequate sleep can partly offset fatigue resulting from heavy training loads. Complementing these findings, Souabni et al. (2024) reported that athletes frequently experience sleep deprivation during academic semesters but tend to achieve more restorative rest during holidays. Similarly, Hrozanova et al. (2019) demonstrated that youth athletes’ sleep patterns are influenced by psychological stress, training demands, and social support structures. Collectively, this body of evidence underscores sleep as an indispensable factor in sustaining performance, supporting mental health, and promoting recovery among adolescent athletes. However, much of the existing research is confined to single-sport or short-term contexts, limiting insights into inter-sport differences and the longitudinal evolution of sleep behaviors. Recent narrative syntheses (Hatia et al., 2024) have also emphasized the need for improved sleep monitoring and targeted interventions across youth sport settings. Moreover, findings concerning the moderating role of sleep in the relationship between emotional intelligence (EI) and athlete burnout remain scarce and sometimes contradictory. Future investigations should therefore adopt multi-sport and longitudinal approaches to clarify these mechanisms and to build a stronger empirical foundation for enhancing psychological well-being and guiding evidence-based recovery strategies in youth sport.

### 2.2 Contributions and limitations of research on sleep, fatigue, and mental health

An expanding body of empirical work has highlighted the close connection between sleep and mental health in adolescent athletes. In a recent study, Choi et al. (2025) found that poorer sleep quality was strongly associated with depressive symptoms among elite youth athletes in South Korea, suggesting that sleep disturbances may act as an early indicator of psychological distress. Rasmussen et al. (2023) likewise reported that adverse childhood experiences heightened the likelihood of sleep difficulties in collegiate athletes, which in turn undermined their mental health. Extending these observations, Silva et al. (2020) identified a clear association between sleep complaints and musculoskeletal injuries, implying that inadequate sleep may compound both psychological strain and physical fatigue, ultimately increasing injury risk. These results are consistent with earlier evidence linking insufficient sleep to higher reports of anxiety and depression among youth athletes (Stracciolini et al., 2019). Building on this evidence, a number of studies have explored how sleep disturbances interact with psychological fatigue. Gomes et al. (2017) showed that sleep quality among adolescent athletes was closely related to symptoms of anxiety and depression, while Yan and Wang (2025) described a mediating pathway in which poor sleep indirectly contributed to health deterioration through elevated psychological stress. Similarly, King et al. (2024) observed that fluctuations in sleep quality during competitive periods were directly reflected in young soccer players’ perceived health and emotional well-being. Coel et al. (2023) further emphasized that sleep should be treated as both a predictor and a barometer of mental resilience in youth sport. Collectively, these findings illustrate that sleep functions not only as a physiological necessity but also as a key determinant of mental resilience in sport. Despite these contributions, several important limitations remain. Much of the available research relies on cross-sectional designs, limiting causal inferences between sleep and mental health (Wilson et al., 2025). In addition, participant samples often originate from single nations or sporting contexts, restricting the generalizability of results across cultures and disciplines (Predoiu et al., 2024). Furthermore, Valier et al. (2017) demonstrated substantial sport- and sex-based differences in fatigue and quality-of-life scores, underscoring the need for more differentiated baseline data. Another notable shortcoming is that few studies have integrated emotional intelligence (EI) into their analytical frameworks, even though EI may play a regulatory role in buffering the effects of sleep disruption and psychological fatigue. Neglecting this variable leaves a gap in understanding how emotional regulation mechanisms interact with recovery processes. Encouragingly, recent scholarship has begun to take a broader and more integrated perspective. Wilson et al. (2025) argued that sleep health should be conceptualized within a holistic model of athlete development encompassing emotional regulation, recovery, and training load. In addition, emerging evidence from Xue et al. (2024) suggests that EI significantly shapes adolescents’ psychological responses to sport-related stress, highlighting the need to integrate EI into models of sleep and recovery. Yet, systematic empirical studies examining the dynamic relationships among EI, sleep, and mental health in adolescent athletes remain scarce. Addressing this gap would help construct a more nuanced understanding of the psychological processes that sustain recovery and well-being in youth sport—and, in turn, inform more effective interventions for athlete mental health.

### 2.3 Training load, sport-type differences, and mechanisms of burnout

Training load and sport type are widely recognized as critical factors shaping patterns of sleep, fatigue, and burnout in adolescent athletes. Prolonged exposure to intensive training environments has consistently been associated with a greater risk of sleep disturbance and symptoms of overtraining. For example, Cho and Im (2024) observed that young athletes who reported disrupted sleep often displayed signs of overtraining, a condition that appeared to heighten both physical exhaustion and psychological strain. In a related line of inquiry, Aloulou et al. (2021) highlighted that training load and sport discipline exert distinct effects on sleep, as each sport places unique physiological and psychological demands on athletes’ recovery routines. Meta-analytic evidence also shows that sport-specializing adolescents face higher burnout risks than those who sample multiple sports (Giusti et al., 2020), reinforcing the importance of sport-type differences. Seasonal dynamics further complicate this picture. Chou et al. (2021) noted that during competitive phases, elite basketball players tended to experience reduced sleep quality alongside elevated stress, underscoring how the intensity of competition can undermine rest and restoration. Evidence from longitudinal comparisons also reflects how external factors reshape these relationships. Drawing on pre- and post-pandemic data, Wingerson et al. (2021) found that shifts in training settings and competitive schedules significantly altered young athletes’ sleep patterns, activity levels, and perceived quality of life. Training load does not only affect physiological recovery—it may also influence psychological health indirectly through its effect on sleep. Watson and Brickson (2018) demonstrated that sleep disruption mediated the link between training demands and subjective well-being, illustrating a nuanced mechanism connecting workload, rest, and emotional functioning. More recent findings by Tate et al. (2025a) reinforced this pattern, showing a pronounced negative association between heavy training loads and sleep quality. Complementary evidence from Tate et al. (2025b) further revealed that inadequate sleep impairs cognitive functioning in adolescent athletes, with consequences that extend beyond sport to academic performance. Research on burnout has also begun to consider sport-type and demographic factors. Schorb et al. (2023) and Billa et al. (2023) demonstrated substantial variability in burnout levels across different sports, whereas Xue et al. (2024) highlighted EI’s central role in shaping burnout among adolescent basketball players. These findings underscore that both sport characteristics and intra-personal factors interact to influence burnout trajectories. Taken together, these findings reveal a complex web of interactions among training intensity, sport-specific demands, and recovery processes. Yet, current research still faces notable gaps. Most available studies concentrate on a single gender or focus narrowly on one sport, limiting broader applicability. As Aloulou et al. (2021) suggest, future investigations would benefit from cross-sport and mixed-gender approaches to better capture the diversity of athletes’ experiences and to clarify the mechanisms underlying burnout.

## 3 Methods

### 3.1 Research design and participants

Eligible participants were full-time university students aged 18–24 years who had engaged in organized team or individual training and competitions at the university or professional level within the previous 12 months and provided informed consent to participate voluntarily. Athletes were excluded if they had sustained serious injuries preventing regular training or competition or had been diagnosed with psychiatric or neurological disorders. Ethical approval for the study was obtained from the institutional review boards of the participating universities, and all procedures conformed to the principles of the Declaration of Helsinki. Participants were informed of their rights, and voluntary participation, confidentiality, and data anonymity were ensured throughout the research process (Table 1).

**Table 1:** Demographic characteristics of the participants (*N* = 450).

Variable	Value
Total sample	450
Team sports	213 (47.3%)
Individual sports	237 (52.7%)
Mean age (years)	20.01
Age SD	1.09
Age range	18–23

### 3.2 Measurement instruments

All core variables were measured using standardized instruments with well-established reliability and validity in sport psychology and sport medicine research, ensuring the rigor of the assessment process. Emotional intelligence (EI) was assessed using a combined version of the Wong and Law Emotional Intelligence Scale (WLEIS; Wong and Law, 2002) and the Schutte Self-Report Inventory (SSRI; Schutte et al., 2001). The 16 items cover four domains—self-emotion appraisal, others’ emotion appraisal, emotion regulation, and emotion management—and are rated on a 7-point Likert scale (1 = strongly disagree, 7 = strongly agree), with higher scores indicating greater EI. Sleep quality was evaluated through the Pittsburgh Sleep Quality Index (PSQI; Buysse et al., 1989) together with an athlete-specific questionnaire assessing sleep latency, continuity, depth, and perceived restoration; higher scores reflected poorer sleep quality. Fatigue was measured using the Fatigue subscale of the Profile of Mood States (POMS; McNair et al., 1971), which captures both physical and mental aspects such as low energy, sleepiness, depressed mood, and reduced motivation. Responses were recorded on a 5-point Likert scale, where higher scores indicated greater fatigue. Delayed onset muscle soreness (DOMS) was assessed using the Muscle Soreness Visual Analogue Scale (VAS; Cheung et al., 2003), ranging from 0 (“no soreness”) to 10 (“extreme soreness”), with higher values denoting greater perceived muscle soreness. Finally, athletic burnout was measured with the Athlete Burnout Questionnaire (ABQ; Raedeke and Smith, 2001), which includes three dimensions—emotional exhaustion, reduced sense of accomplishment, and sport devaluation—rated on a 5-point Likert scale (1 = strongly disagree, 5 = strongly agree). Higher total scores represented a higher level of burnout.

### 3.3 Data collection and data screening

Data were collected both online and offline. For the offline sessions, the research team collaborated with university departments of physical education and varsity teams. Research assistants distributed printed questionnaires during training breaks or after class, gave brief standardized instructions, and collected the completed forms on site. Online data were gathered using the “Questionnaire Star” platform, and the survey link was shared through official university channels, including varsity team WeChat groups and departmental announcements. Each device was limited to a single submission, and the system required all items to be completed before the questionnaire could be submitted. At the beginning of the survey, participants were informed of the study’s purpose, the anonymous treatment of their responses, and that all data would be used solely for academic research. They were asked to complete the questionnaire independently, without guidance from coaches or teammates.

Questionnaires were screened for quality before analysis. Responses were excluded if the completion time was unrealistically short (<120 s), if answer patterns showed inconsistencies or uniform selections, if duplicate entries were detected based on IP address or device number, or if data were missing for key variables. In total, 486 questionnaires were collected. After removing 36 invalid cases, 450 valid responses were retained for statistical analysis.

### 3.4 Data analysis

Before statistical testing, all data were anonymized to protect participant confidentiality. Initially, Cronbach’s *α*, composite reliability (CR), and average variance extracted (AVE) were computed to assess the internal consistency and convergent validity of the study measures. Pearson correlation analyses were then performed to examine the associations among emotional intelligence (EI), sleep quality, fatigue, delayed onset muscle soreness (DOMS), and athletic burnout. Multiple regression analyses were conducted to test the main effects of sleep quality, fatigue, and DOMS on burnout, followed by additional models including interaction terms (EI × Sleep, EI × Fatigue, and EI × DOMS) to evaluate the moderating role of EI. Multi-group structural equation modeling (SEM) was applied to compare path coefficients between team and individual sport athletes. Finally, bootstrap resampling with 5,000 iterations was used to estimate confidence intervals and test the significance of all paths. All statistical tests were two-tailed, and the significance level was set at *p* < 0.05.

### 3.5 Measurement framework of core study variables

**Figure 2:**
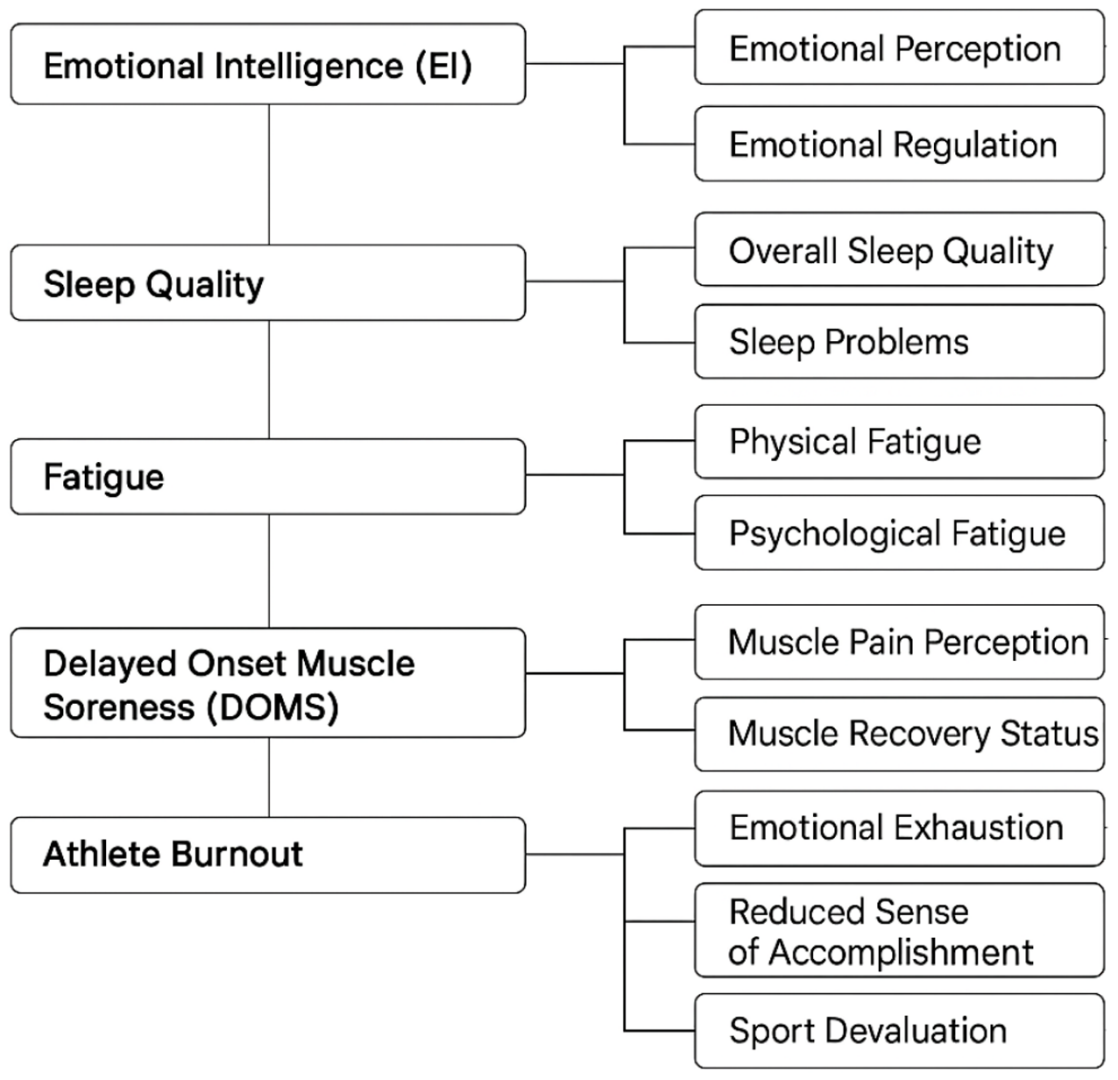
Measurement framework of core study variables.

Figure 2 shows measurement framework of core study variables.

## 4 Results

### 4.1 Measurement reliability and validity

Table 2 summarizes the reliability and validity results for the study measures. All core variables showed good internal consistency, with Cronbach’s α coefficients exceeding 0.85. Specifically, Cronbach’s α values were 0.89 for emotional intelligence, 0.87 for sleep quality, 0.88 for fatigue, 0.85 for DOMS, and 0.96 for athletic burnout. All exceeded the recommended threshold of 0.70, indicating strong reliability across measures. Composite reliability (CR) values were all above 0.85, supporting the robustness of the measurement scales. For convergent validity, the average variance extracted (AVE) values ranged from 0.57 (DOMS) to 0.68 (burnout), with all constructs exceeding the 0.50 threshold, indicating adequate convergent validity. Overall, the measurement instruments met established psychometric standards, supporting their suitability for subsequent structural equation modeling analyses.

**Table 2:** Reliability and validity test results.

Variable	Cronbach’s *α*	AVE	CR
Emotional intelligence (EI)	0.89	0.61	0.87
Sleep quality (Sleep)	0.87	0.59	0.86
Fatigue	0.88	0.62	0.88
DOMS	0.85	0.57	0.85
Athlete burnout	0.96	0.68	0.92

### 4.2 Comparison of emotional intelligence between team and individual athletes

An independent-samples t test showed a significant difference in emotional intelligence (EI) between team- and individual-sport athletes (Table 3). Team-sport athletes scored higher on EI (*M* = 5.31, SD = 0.60) than those in individual sports (*M* = 4.70, SD = 0.60), *t*(448) = 25.91, *p* < 0.001. This result supports the contextual socialization theory, suggesting that the social environment in which athletes train and compete plays an important role in shaping emotional competencies. In team sports, where cooperation and interpersonal interaction are frequent, athletes have more opportunities to recognize, express, and regulate emotions, which may enhance their emotional intelligence. By contrast, individual sports often involve self-directed practice and performance with fewer opportunities for social feedback, potentially limiting the development of emotional skills. These findings highlight how sport contexts can shape psychosocial development and point to the need for tailored approaches in athlete training and support. For team-sport athletes, group-based emotional training and peer interaction may strengthen EI, whereas for individual-sport athletes, targeted counseling and emotion management programs could help compensate for reduced social engagement.

**Table 3:** EI differences between team and individual athletes.

Group	M	SD
Team athletes	5.31	0.6
Individual athletes	4.7	0.6
*t*-value	25.91	
*p*-value	<0.001***	

*p < 0.05, **p < 0.01, ***p < 0.001.

### 4.3 Correlation analysis among variables

Correlation analyses revealed several noteworthy relationships among the main variables (Table 4). Athletes with higher emotional intelligence (EI) tended to report better sleep quality (*r* = 0.15, *p* < 0.01) and lower levels of burnout (*r* = −0.20, *p* < 0.001). The association between EI and fatigue, however, was negligible (*r* = −0.02, n.s.), implying that EI exerts little influence on short-term physiological tiredness. Better sleep was also linked to reduced burnout (*r* = −0.20, *p* < 0.001), highlighting the restorative role of sleep in protecting athletes from psychological exhaustion. By contrast, both fatigue (*r* = 0.25, *p* < 0.001) and delayed onset muscle soreness (DOMS) (*r* = 0.39, *p* < 0.001) were positively related to burnout, suggesting that physical strain and soreness arising from intensive training may accelerate mental depletion. A small but significant negative link was also observed between EI and DOMS (*r* = −0.08, *p* < 0.05), indicating that emotionally skilled athletes might cope more effectively with discomfort or pain. Taken together, these findings suggest that while EI contributes meaningfully to psychological resilience, its role in moderating direct physical load appears limited. Further work could use longitudinal designs to test whether EI buffers the effects of physical strain indirectly—for example, by promoting better sleep or reducing perceived stress—rather than serving as a universally protective psychological trait.

**Table 4:** Correlation matrix (*N* = 450).

Variable	EI	Sleep	Fatigue	DOMS	Burnout
EI	1	0.15**	−0.02	−0.08*	−0.20***
Sleep	0.15**	1	0.03	−0.07*	−0.20***
Fatigue	−0.02	0.03	1	0.02	0.25***
DOMS	−0.08*	−0.07*	0.02	1	0.39***
Burnout	−0.20***	−0.20***	0.25***	0.39***	1

*p < 0.05, **p < 0.01, ***p < 0.001.

### 4.4 Main effects of sleep, fatigue, and DOMS on athlete burnout

Regression analyses (Table 5) showed that sleep quality, fatigue, and delayed onset muscle soreness (DOMS) each contributed significantly to variations in athlete burnout. Better sleep was associated with lower burnout scores (*β* = −0.21, *t* = −4.48, *p* < 0.001), suggesting a protective role of adequate rest against psychological exhaustion. Fatigue (*β* = 0.31, *t* = 6.10, *p* < 0.001) and DOMS (*β* = 0.31, *t* = 9.03, *p* < 0.001), by contrast, were both positively linked to burnout, implying that the physical toll of intensive training—manifested as tiredness and muscle soreness—can erode athletes’ mental resources. These patterns indicate that improving sleep alone may be insufficient to offset the effects of heavy training loads; under high-intensity conditions, physical strain may quickly translate into mental fatigue. In practice, managing training demands together with recovery strategies, and supplementing them with psychological support or emotion-regulation training, may offer a more effective way to limit burnout risk. Taken together, the findings reinforce the close connection between athletes’ physical state and psychological exhaustion and point to actionable directions for evidence-based training and mental health programs for youth athletes.

**Table 5:** Regression analysis of sleep, fatigue, and DOMS on burnout.

Variable	*β*	*t*-value	*p*-value
Sleep	−0.21	−4.48	<0.001***
Fatigue	0.31	6.1	<0.001***
DOMS	0.31	9.03	<0.001***

*p < 0.05, **p < 0.01, ***p < 0.001.

### 4.5 Moderating effect of emotional intelligence

**Figure 3:**
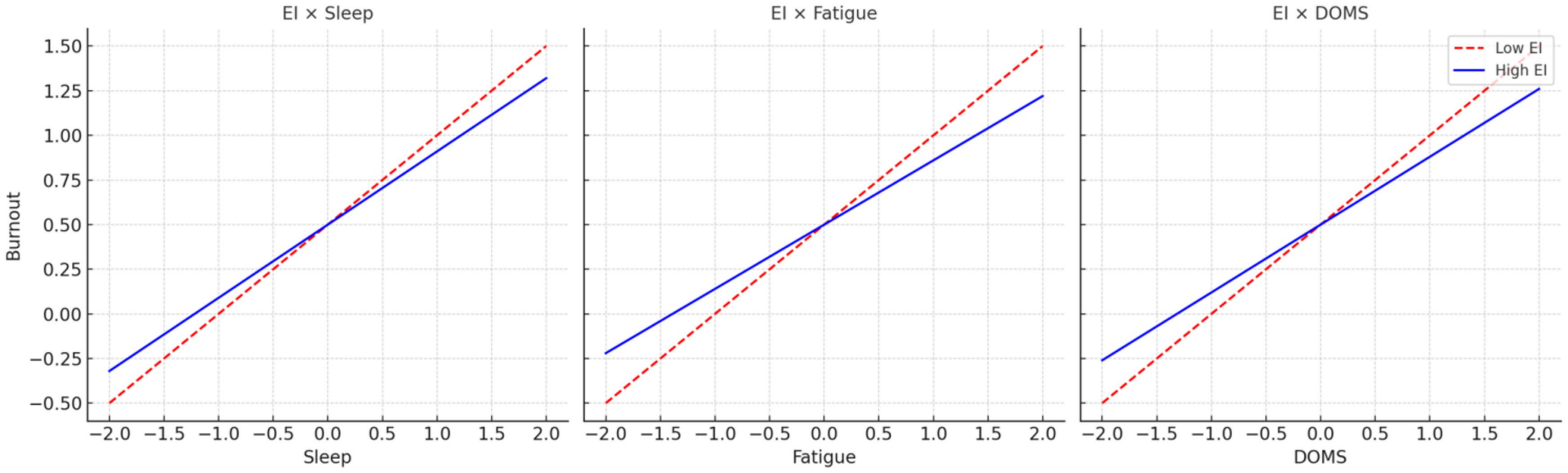
Interaction effects of emotional intelligence (EI) with sleep quality, fatigue, and delayed onset muscle soreness (DOMS) on athlete burnout.

Moderation analyses (Table 6; Figure 3) identified emotional intelligence (EI) as a key factor influencing how youth athletes adapt psychologically to training demands. EI altered the strength of the associations between sleep quality, fatigue, delayed onset muscle soreness (DOMS), and burnout. Higher EI reduced the negative impact of poor sleep on burnout (*β* = −0.09, *t* = −3.21, *p* = 0.001): when EI was low, lack of sleep tended to heighten feelings of exhaustion, whereas this effect was markedly weaker among athletes with higher EI. A similar pattern emerged for fatigue (*β* = −0.14, *t* = −2.96, *p* = 0.003); athletes with stronger emotional skills showed slower increases in burnout despite experiencing comparable levels of physical tiredness. EI also mitigated the positive link between DOMS and burnout (*β* = −0.12, *t* = −2.78, *p* = 0.005), suggesting that even when muscle soreness rises after intensive training, emotionally intelligent athletes are better able to stay balanced—likely through more adaptive emotional regulation and cognitive reframing. Overall, the results indicate that EI functions as a psychological buffer within athletic settings, while underscoring a limitation of training programs that emphasize physical conditioning but devote less attention to emotional development. In applied terms, improving sleep hygiene or reducing training volume alone is unlikely to prevent burnout. Incorporating EI enhancement into athlete development may provide a more sustainable approach to resilience building and long-term well-being in competitive sport.

**Table 6:** Moderating effect of EI.

Interaction term	*β*	t-value	p-value
EI × Sleep	−0.09	−3.21	0.001**
EI × Fatigue	−0.14	−2.96	0.003**
EI × DOMS	−0.12	−2.78	0.005**

*p < 0.05, **p < 0.01, ***p < 0.001.

### 4.6 Multi-group analysis (team vs. individual sports)

**Figure 4:**
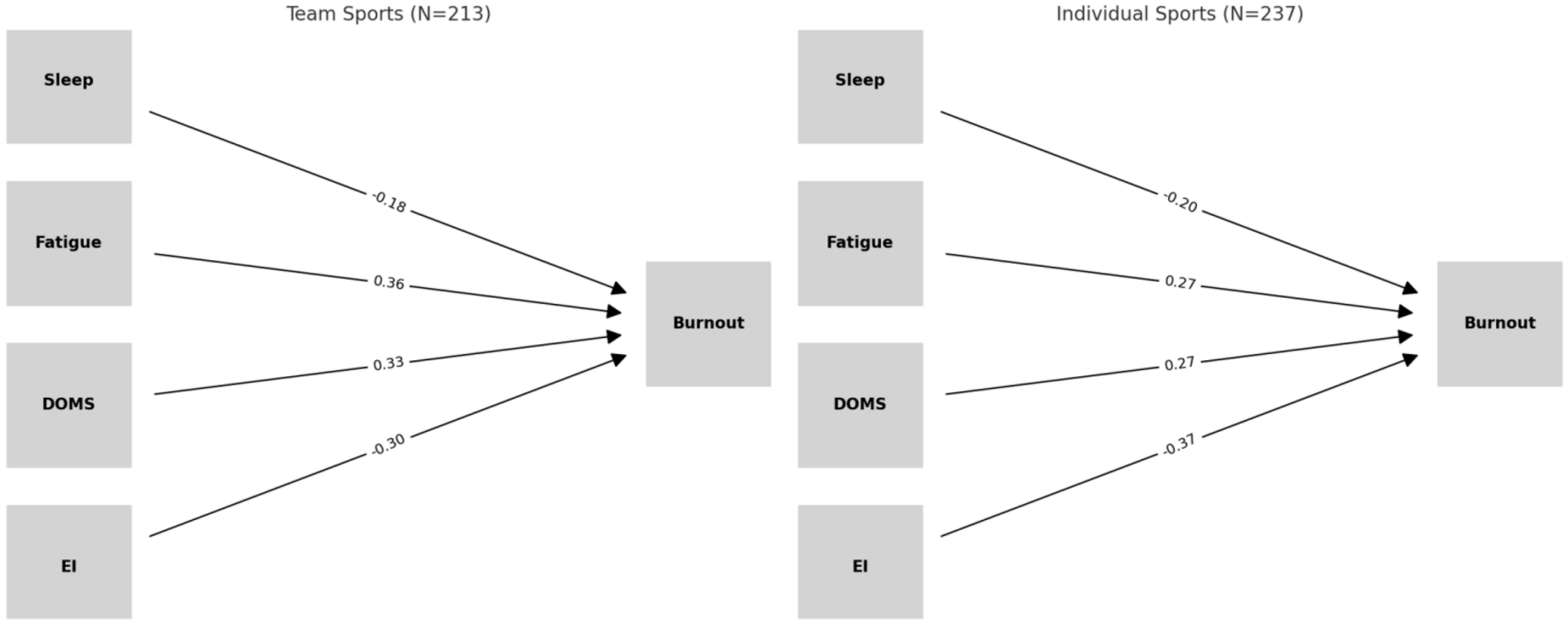
Multi-group structural equation modeling (SEM) path analysis comparing team sports (*N* = 213) and individual sports.

Multi-group path analysis identified clear differences between team- and individual-sport athletes in the structural pathways associated with burnout (Table 7; Figure 4). Sleep quality showed a negative relationship with burnout in both groups, and the effect was stronger among individual-sport athletes (team: *β* = −0.18, *p* < 0.01; individual: *β* = −0.20, *p* < 0.01). This finding suggests that insufficient restorative sleep more severely impairs athletes who train and compete without the support of a team. Fatigue demonstrated a stronger positive association with burnout among team-sport athletes (*β* = 0.36, *p* < 0.001) than among individual-sport athletes (*β* = 0.27, *p* < 0.001), which is consistent with the collective, high-intensity training routines common in team settings where accumulated fatigue tends to develop more rapidly. A comparable trend was observed for delayed onset muscle soreness (DOMS), with stronger effects in team sports (*β* = 0.33, *p* < 0.001) than in individual sports (*β* = 0.27, *p* < 0.001). This suggests that inadequate recovery management in team-based environments may exacerbate post-training psychological exhaustion. In contrast, emotional intelligence (EI) showed a stronger negative association with burnout among individual-sport athletes (team: *β* = −0.30, *p* < 0.001; individual: *β* = −0.37, *p* < 0.001). When team-based support is limited, athletes appear to depend more on self-regulation to manage the psychological strain of training and competition. This result highlights that the protective role of EI differs according to the social and structural context of the sport. Practically, these results indicate that the training environment plays a defining role in shaping the development of burnout. For team sports, competitive success should be balanced with systematic recovery and load management to prevent excessive physical and psychological strain. For individual sports, the stronger protective value of EI points to the need for integrating psychological-skills training and emotional intelligence development into athlete preparation, particularly when peer interaction is minimal. The findings also invite reflection on current youth training paradigms, which often emphasize physical conditioning and performance outcomes over the development of psychological resources. This imbalance may weaken resilience over time and shorten athletic career longevity. By comparing athletes across sport types, the present study clarifies distinct mechanisms contributing to burnout and offers empirical evidence for sport-specific psychological interventions and differentiated training strategies.

**Table 7:** Path differences between team and individual athletes.

Path	Team sports (*β*)	Individual sports (*β*)	Group difference
Sleep → Burnout	−0.18**	−0.20**	Significant
Fatigue → Burnout	0.36***	0.27***	Significant
DOMS → Burnout	0.33***	0.27***	Significant
EI → Burnout	−0.30***	−0.37***	Significant

*p < 0.05, **p < 0.01, ***p < 0.001.

### 4.7 Robustness and auxiliary analyses

To ensure the robustness of the findings, multiple reliability tests were conducted (Table 8). First, the bootstrap analysis indicated that the 95% confidence intervals for the effects of sleep quality (95% CI = [−0.29, −0.08]), fatigue (95% CI = [0.22, 0.40]), delayed onset muscle soreness (DOMS; 95% CI = [0.23, 0.37]), and emotional intelligence (EI; 95% CI = [−0.45, −0.19]) on burnout did not include zero, confirming the statistical significance and stability of the core path relationships. Second, a robustness check using an alternative indicator of sleep (Sleep 1) yielded consistent results, with sleep remaining a significant negative predictor of burnout (*β* = −0.18, *p* < 0.01), suggesting that the protective effect of sleep quality is not measurement-specific. Finally, sensitivity analyses demonstrated that the main results remained stable across gender and academic-year subgroups, supporting the generalizability and external validity of the conclusions. Collectively, these results affirm the robustness of the proposed model and reinforce the central argument of this study—that athlete burnout among youth is shaped by the joint influence of multiple factors, with EI serving as a critical protective mechanism. Practically, the consistency of these findings across subsamples and analytic approaches indicates that the observed relationships are not sample-dependent or instrument-specific but rather reflect generalizable psychological and physiological processes. The proposed integrative model—linking sleep quality, fatigue, physical load, and emotional intelligence in predicting burnout—thus demonstrates cross-group applicability and underscores the importance of incorporating EI development into youth athlete training and development programs.

**Table 8:** Robustness tests.

Test type	Result
Bootstrap (95% CI)	Sleep [−0.29, −0.08]; Fatigue [0.22, 0.40]; DOMS [0.23, 0.37]; EI [−0.45, −0.19] → all CIs exclude zero
Substitution test	Sleep1 → Burnout: *β* = −0.18, *p* < 0.01
Sensitivity analysis	Results consistent across gender and grade levels

## 5 Discussion

### 5.1 Physiological load and burnout: clarifying the dual effects of sleep, fatigue, and DOMS within existing stress and recovery models

The findings indicate that sleep quality plays a meaningful protective role in the development of burnout among adolescent athletes, whereas training fatigue and delayed-onset muscle soreness (DOMS) emerge as more powerful risk factors whose effects surpass those of sleep. This pattern reveals a persistent imbalance between the physiological demands placed on young athletes and the recovery resources available to them. Although adequate sleep can ease psychological exhaustion to some extent, it is insufficient to counteract the cumulative strain produced by intensive training. Consistent with the core assumptions of stress and recovery balance models, the present results further suggest that, given their ongoing physical maturation and developing psychological regulatory capacities, adolescents are particularly vulnerable when adult-oriented training expectations are imposed on them. When performance-driven training structures offer limited or inconsistent recovery opportunities, fatigue is likely to accumulate more rapidly and heighten the risk of psychological depletion. In many youth sport settings, improving sleep is often regarded as the simplest intervention; however, the evidence from this study shows that, without continuous monitoring of training load and more deliberate, structured recovery planning, sleep enhancement alone is unlikely to slow or prevent the progression of burnout. High training loads paired with compressed recovery periods remain common practice, and although such approaches may support short-term performance gains, they frequently come at the cost of declining mental health and early-stage burnout. To build a more sustainable training environment for youth athletes, equal attention must be paid to sleep quality, fatigue accumulation, and muscular recovery, accompanied by a critical reassessment of performance-centered institutional norms. Achieving a more balanced alignment between physical demands and recovery resources is essential not only for supporting long-term athletic development but also for safeguarding the psychological well-being of young athletes during a sensitive developmental period.

### 5.2 Emotional intelligence as a psychological buffer: its independent role in protecting youth athletes from burnout

The findings of this study highlight the distinctive and essential role of emotional intelligence (EI) as a psychological buffer that mitigates the impact of multiple physiological stressors on burnout among adolescent athletes. The interaction analyses showed that EI attenuates the effects of insufficient sleep, accumulated fatigue, and delayed-onset muscle soreness (DOMS), suggesting that athletes with higher EI are better able to maintain psychological stability under sustained physical and competitive demands. This pattern reflects the unique importance of EI during adolescence—a developmental period in which emotional regulation and cognitive control systems are still maturing—and indicates that EI provides forms of psychological protection that cannot be substituted by sleep or other recovery-based mechanisms alone. By enabling athletes to interpret training-related stress more adaptively and manage negative emotions more effectively, EI offers an internal resource that remains influential even when external recovery conditions are limited or inconsistent. This is particularly relevant for young athletes, who often operate with restricted autonomy, limited emotional support, and uneven recovery opportunities, making them more reliant on internal psychological capacities to prevent physical strain from escalating into psychological exhaustion. The present study contributes to the literature by demonstrating that EI continues to exert protective effects even in contexts of inadequate recovery, thereby filling a gap in existing burnout research that has often focused on physical or environmental factors while overlooking the mechanisms through which internal resources shape stress adaptation. The findings also point to broader structural issues within youth sport systems, where physical conditioning and technical development frequently receive far greater emphasis than psychological skill-building. When EI and other internal capacities are neglected, athletes face cumulative pressures—poor sleep, persistent fatigue, recurrent soreness—without the psychological buffers needed to withstand them, substantially increasing the risk of burnout. Recovery-focused interventions, although valuable, are unlikely to prevent long-term maladaptation if they are not paired with intentional EI development. By identifying EI as a key link between physiological load and psychological fatigue, this study not only advances theoretical understanding of youth athlete burnout but also provides practical justification for integrating emotional competence training alongside load management and recovery planning. Embedding EI development within training structures can help young athletes sustain both performance and psychological resilience, supporting healthier and more stable long-term involvement in sport.

### 5.3 Context-specific burnout pathways across sport types: implications for training environments and psychological resource development

The multi-group analysis revealed distinct burnout pathways across different sport types, indicating that the mechanisms linking physiological strain and psychological exhaustion are shaped by the training environments in which athletes participate. Sleep quality was negatively associated with burnout in both team and individual sports, yet its influence was more pronounced among athletes who train or compete independently. For these individuals, sleep appears to function as a primary recovery mechanism, likely because they must rely more heavily on internal regulation in the absence of continuous interpersonal support. In contrast, fatigue and delayed-onset muscle soreness (DOMS) showed stronger positive effects on burnout among team-sport athletes, reflecting how the cumulative strain of collective, high-intensity training can extend beyond physical discomfort and contribute to psychological exhaustion. Emotional intelligence (EI) served as a protective factor in both sport types, but its buffering effect was stronger among individual-sport athletes. This pattern aligns with the logic of contextual socialization theory: the social and structural features of training environments not only shape behavioral demands but also influence how psychological resources are activated during stress. In team sports, shared goals and group pressure often encourage cohesion and collaboration, yet when training loads are not managed appropriately, accumulated physical fatigue can rapidly translate into psychological strain. In individual sports, athletes must depend more on self-regulation to handle performance demands and the relative isolation of their training context, which makes EI particularly critical for maintaining emotional stability and resilience. These findings underscore a significant limitation of many youth sport programs, where physical training and psychological interventions are often implemented uniformly without regard to the contextual differences between sport types. Such uniformity creates mismatches between the demands athletes face and the resources available to them, thereby increasing their vulnerability to burnout. Developing sport-specific intervention strategies is therefore essential. Team-sport programs may benefit from more rigorous monitoring of training load and structured recovery systems, while individual-sport environments should emphasize systematic EI development and provide consistent psychological support to offset the limited social interaction inherent in these settings. Tailoring training and psychological support to the distinctive features of each sport type can help safeguard long-term mental health while supporting sustained athletic performance and healthier developmental trajectories in youth athletes.

## 6 Conclusion

This study shows that burnout in youth athletes does not arise from a single source of strain but develops through the long-term interaction between physiological load and psychological resources. Sleep quality played a consistent protective role, whereas training fatigue and delayed-onset muscle soreness (DOMS) substantially increased the likelihood of psychological exhaustion. Emotional intelligence (EI) moderated these effects by reducing the impact of physiological stress, demonstrating that EI is not a peripheral trait but a psychological resource that helps young athletes maintain emotional stability and adapt to training demands. The pathways also differed across sport types. Team-sport athletes were more vulnerable to the cumulative strain produced by collective, high-intensity training environments, while athletes in individual sports relied more on sleep and emotional regulation to sustain psychological balance. These findings extend existing burnout models by incorporating a joint physiological–psychological perspective and underline the need for training systems that support not only physical and technical development but also the cultivation of psychological competencies. Adjusting recovery routines alone is unlikely to reduce burnout risk in the long term; effective prevention requires coordinated management of training load, EI development, and sport-specific support structures. Although the study is limited by its cross-sectional design and relatively homogeneous sample, the findings provide a useful foundation for building youth sport environments that balance performance development with long-term mental well-being.

### 6.1 Limitations and future directions

Recent studies have explored a range of interventions targeting sleep and recovery among youth athletes. Luetmer et al. (2019), in research with adolescent soccer players, found that acupuncture not only reduced delayed onset muscle soreness (DOMS) but also enhanced athletes’ perceived well-being, indicating the potential of traditional rehabilitation techniques in supporting recovery. Mason et al. (2025) observed strong interconnections among sleep, nutrition, and recovery in elite youth athletes; balanced dietary strategies combined with structured recovery routines improved sleep quality, alleviated fatigue, and supported mental health. Wingerson et al. (2021) further reported that, following the COVID-19 pandemic, athletes who trained at home and faced fewer competitions slept longer and experienced better life quality and activity levels. Together, these findings provide useful guidance for adjusting training intensity and scheduling to improve athlete recovery. Despite these advances, the current literature still faces several methodological and conceptual limitations. Many studies involve small samples and short intervention periods, which restrict the ability to infer causal relationships (Mason et al., 2025). Moreover, most interventions have concentrated on physiological recovery while overlooking psychological aspects—particularly the role of emotional intelligence (EI) in reducing fatigue, improving sleep, and preventing burnout (Wilson et al., 2025). Existing work has also tended to focus on specific sports or single cultural settings, thereby constraining the generalizability and external validity of results. Future research would benefit from integrating psychological and physiological perspectives to strengthen methodological rigor and applied relevance. Longitudinal and experimental designs are needed to clarify the dynamic interactions among sleep, fatigue, EI, and burnout. Expanding samples to include athletes of different genders, age groups, and cultural backgrounds would also enhance representativeness and external validity. Incorporating multiple data sources—such as wearable devices, physiological indicators, and validated psychological scales—could further improve the objectivity and ecological validity of assessments. It would also be valuable to examine how contextual factors, including team climate, social support, and training culture, influence recovery processes, as well as to assess the long-term impact of integrated interventions on mental health, performance, and career longevity. Developing a comprehensive model that links sleep management, emotional intelligence, nutrition, and psychological recovery could ultimately inform more coherent and sustainable approaches that promote both athletic achievement and overall well-being among youth athletes.

## Data Availability

The raw data supporting the conclusions of this article will be made available by the authors, without undue reservation.
